# Stem Cell Exosomes for Osteoarthritis in Veterinary Medicine

**DOI:** 10.1155/sci/4888569

**Published:** 2025-07-16

**Authors:** S. Amitha Banu, Khan Sharun, Rony S. Emmanuel, Merlin Mamachan, K. M. Manjusha, Sathish Muthu, Hussein M. El-Husseiny, Rohit Kumar, Abhijit M. Pawde, Kuldeep Dhama, Amarpal

**Affiliations:** ^1^Division of Surgery, ICAR-Indian Veterinary Research Institute, Izatnagar, Bareilly, Uttar Pradesh, India; ^2^Graduate Institute of Medicine, Yuan Ze University 32003, Taoyuan, Taiwan; ^3^Division of Physiology and Climatology, ICAR-Indian Veterinary Research Institute, Izatnagar, Bareilly, Uttar Pradesh, India; ^4^Department of Orthopaedics, Orthopaedic Research Group, Coimbatore 641045, Tamil Nadu, India; ^5^Central Research Laboratory, Meenakshi Medical College Hospital and Research Institute, Meenakshi Academy of Higher Education and Research, Kanchipuram 631552, Tamil Nadu, India; ^6^Institute of Global Innovation Research, Tokyo University of Agriculture and Technology, Fuchu-shi, Tokyo, Japan; ^7^Department of Surgery, Anesthesiology and Radiology, Faculty of Veterinary Medicine, Benha University, Moshtohor, Toukh, Elqaliobiya 13736, Egypt; ^8^Laboratory of Veterinary Physiology, Department of Veterinary Medicine, Tokyo University of Agriculture and Technology, Tokyo, Japan; ^9^Division of Pathology, ICAR-Indian Veterinary Research Institute, Izatnagar, Bareilly, Uttar Pradesh, India

**Keywords:** cartilage regeneration, cell-free therapy, exosomes, extracellular vesicles, osteoarthritis, veterinary medicine

## Abstract

Osteoarthritis (OA) is a growing health concern worldwide. This disease is a major concern in human and veterinary patients, especially in growing and geriatric individuals. The poor regenerative capacity of damaged cartilage affects the healing process. Currently, no effective treatment strategy exists that provides a complete cure. Despite several traditional and pharmacological treatments, none of them resulted in the repair and regeneration of cartilage tissue. Regenerative therapy has gained increasing attention in the treatment of OA as it is directly involved in the regenerative process of damaged cartilage. The mesenchymal stem cells (MSCs) have therapeutic potential in treating OA resulting from their paracrine action on host cells, mediated via cytokines, exosomes, growth factors, and extracellular matrix molecules. Even though no significant side effects are documented, cell-based therapeutics could still present some risks. Exosomes, on the other hand, act primarily by channelizing the resident cells to restore the damaged cartilage and thus play an essential role in the treatment of OA. This review explores the regenerative efficacy of exosomes in managing OA in veterinary patients, elucidating their mechanisms of action and therapeutic potential. Recognizing the importance of comprehending exosomes and their mechanisms is crucial for developing safe and effective cell-free therapeutics for OA. This paper aims to enhance our understanding of cell-free regenerative strategies, paving the way for the development of innovative treatments for OA in veterinary medicine.

## 1. Introduction

Osteoarthritis (OA) is a common, debilitating disease of joints [1]. OA is a widespread degenerative joint disorder impacting articular cartilage, subchondral bone, and entire joints like the knee and hip, marked by cartilage breakdown, subchondral bone hardening, and synovitis [2]. Even though the prevalence of OA is increasing, clinically effective and economical treatments are unavailable. Moreover, the restoration of compromised cartilage is a challenge of considerable appeal to researchers and clinicians [3]. OA is characterized by reactive new bone growth at the articular edges and articular cartilage degradation, resulting in discomfort and stiffness in the affected joints. All synovial joints can develop OA, but the hip and knee are the most affected, frequently resulting in physical impairment [4]. Although OA is commonly identified in older dogs, it is not a natural aspect of aging. In addition, canine OA is typically a subsequent complication of trauma, such as aberrant loading on a normal joint (such as joint damage) or normal force on an abnormal joint, in contrast to human and feline OA (e.g., elbow and hip dysplasia) [5]. OA is most frequently associated with large breeds of dogs. However, it can affect all breeds of dogs and cats as they age, though severity and time of onset may vary [6]. The etiology of OA is multifactorial. Risk factors such as age, obesity, mechanical injuries, and joint trauma have all been linked to OA [7]. The progression of OA occurs because of the interaction of common variables (injury, misalignment, and improper loading of the joints) and individual factors (old age, female sex, obesity, heredity, and diet), which increases the probability of comorbidity and mortality [8].

The treatment objectives for OA patients include educating the owners on disease management, pain reduction, functional improvement, disability prevention, and altering the disease trajectory. The most important method of managing OA is probably pharmacologic analgesia. Hyaluronic acid (HA) and platelet-rich plasma (PRP) injections into the joints efficiently reduce joint discomfort, increase joint mobility, and affect bone, tendon, muscle, and ligament healing [9]. In addition, PRP boosts anti-inflammatory mediators while decreasing proinflammatory mediators and stimulating chondrogenesis. Alternative OA treatments include physical therapy and weight management [10]. Current treatment approaches have problems with availability, graft rejection risk, and the development of fibrocartilage rather than hyaline cartilage [11–13]. Total joint arthroplasty is a successful treatment for advanced joint diseases; however, the lifespan of prostheses is restricted, and the functional results may be poor [14]. Bedinvetmab (Librela) is a monoclonal antibody approved for managing OA pain in dogs by neutralizing nerve growth factor, a key pain mediator in osteoarthritic joints. It has demonstrated significant efficacy in reducing pain and improving quality of life in clinical studies, with a safety profile showing minimal side effects [15]. Emerging therapies for OA in dogs feature novel mechanisms of action and unique combinations of joint compounds, but there remains a heavy reliance on existing drugs with limited efficacy evidence [16].

Mesenchymal stem cell (MSC)-derived exosomes possess exceptional regenerative capabilities that directly target multiple aspects of OA pathogenesis, including inflammation, cartilage degradation, and impaired tissue repair mechanisms [17]. Unlike exosomes from other cell types, MSC-derived exosomes carry a sophisticated cargo of bioactive molecules specifically designed for tissue regeneration, including growth factors, anti-inflammatory cytokines, and regulatory microRNAs (miRNAs) that can modulate chondrocyte behavior and promote extracellular matrix synthesis [17, 18]. These exosomes demonstrate remarkable chondroprotective effects by promoting the proliferation and migration of chondrocytes while simultaneously inhibiting catabolic enzymes, such as MMP-13 and ADAMTS5 that drive cartilage destruction [18, 19]. The immunomodulatory properties of MSC-derived exosomes are particularly crucial for OA treatment, as they can effectively shift macrophage polarization from the proinflammatory M1 phenotype to the anti-inflammatory M2 phenotype, thereby reducing joint inflammation and creating a more conducive environment for tissue repair [17, 20]. This immunomodulatory capacity is especially important in veterinary applications where chronic inflammation significantly contributes to pain and functional impairment in animals with OA [17]. The versatility of MSC-derived exosomes is further enhanced by their ability to be engineered for enhanced therapeutic properties, allowing for customized treatments based on specific veterinary patient needs [21, 22]. The comprehensive therapeutic effects of MSC-derived exosomes, including their ability to promote angiogenesis, reduce apoptosis, and modulate inflammatory responses, make them uniquely suited for addressing the multifaceted pathology of OA in veterinary patients [17, 23].

This narrative review evaluates the potential of harnessing exosomes as a therapeutic approach for managing OA in veterinary medicine. By exploring the biogenesis, cargo, and mechanisms of action of these extracellular vesicles (EVs), we aim to provide a comprehensive overview of the current state of knowledge in this field. As we navigate the potential benefits and challenges associated with EV therapy, the ultimate goal is to advance its clinical application and improve the quality of life for animals afflicted by OA.

## 2. Pathophysiology of OA

In OA, the cartilage gradually breaks down and wears away, leading to pain, swelling, and decreased joint mobility [24]. The two types of OA are primary and secondary, with the primary form, also known as idiopathic OA, developing in previously healthy joints devoid of any known cause [25]. Aging is a major factor in this type of OA because it causes the cartilage in the joints to break down, which results in an irregular repair mechanism [26, 27]. Secondary OA is mostly due to obesity, repeated trauma or surgery to the joint structures, infection, etc [25, 28].

**Figure 1:**
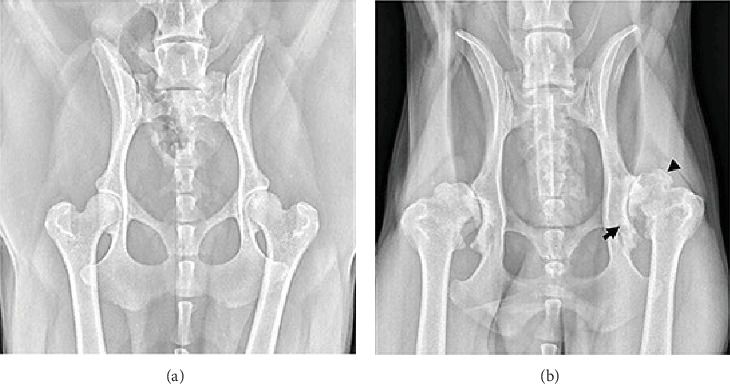
(A) Image of a mature dog displaying radiographically normal hip anatomy. (B) Radiograph from a middle-aged, large-breed male dog showing advanced osteoarthritis (OA) as a consequence of hip dysplasia. Notable features include pronounced new bone development (indicated by the black arrowhead), increased bone density (sclerosis), and structural changes in the acetabulum (black arrow). Reproduced from [29] under the Creative Commons Attribution License (CC BY).

The pathophysiology of OA includes the development of osteophytes (Figure 1), remodeling of the subchondral bone, inflammation of the synovial joint, and articular cartilage degradation, which results in the loss of normal joint function. The chondrocyte is the only type of cell in the articular cartilage extracellular matrix. After cartilage is damaged, the native tissue regenerates with a fibrous structure and lacks the functional properties of intact hyaline cartilage, resulting in reduced functional outcomes and further joint impairment [30]. In addition, inflammation significantly contributes to the development of OA. Various synovial histological responses (proliferation or inflammation) and roentgenographic indications of calcification have been observed in advanced OA [31]. Mechanical OA involves a complex imbalance in cartilage dynamics triggered by joint instability, which, if untreated, can lead to irreversible inflammatory changes like hip dysplasia [32].

**Figure 2:**
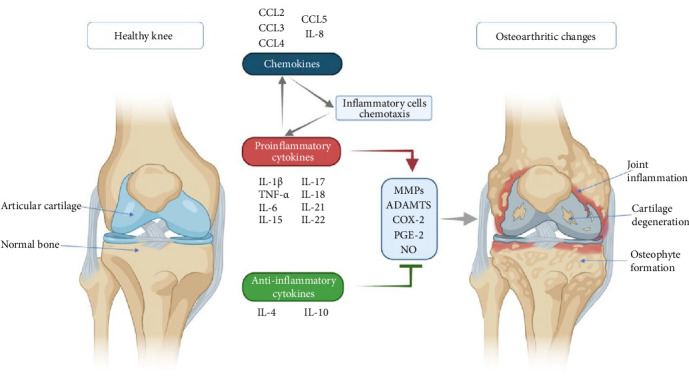
Illustration of the major inflammatory pathways and mediators involved in osteoarthritis (OA) development. An imbalance favoring proinflammatory over anti-inflammatory cytokines promotes the release of catabolic enzymes and inflammatory mediators, contributing to joint tissue damage. This includes structural deterioration, such as cartilage erosion, osteophyte formation, and inflammation of the synovium. Chemokines exacerbate inflammation by attracting immune cells, which in turn amplify the production of proinflammatory cytokines, perpetuating a self-sustaining inflammatory cycle that complicates OA management. IL, interleukin; CCL/CC, chemokine ligand; TNF-α, tumor necrosis factor alpha; MMPs, matrix metalloproteinases; ADAMTS, a disintegrin and metalloproteinase with thrombospondin motifs; COX-2, cyclooxygenase-2; PGE2, prostaglandin E2; NO, nitric oxide. Reproduced from [33] under the terms and conditions of the Creative Commons Attribution (CC BY) license (http://creativecommons.org/licenses/by/4.0/).

An increased number of immune cells with proinflammatory cytokine expressions, such as tumor necrosis factor α (TNF-α) and interleukin (IL)-1β, were found in synovial tissue taken from an OA patient (Figure 2). In addition, extracellular matrix remodeling is directly attributed to the actions of matrix metalloproteinases (MMPs) 1, 3, and 13 [34]. None of the currently available pharmacologic treatments has scientifically proven structure-modifying efficacy [35]. When nonsurgical therapy is ineffective, surgery is preferred. Knee replacement implants also have a finite lifespan and may eventually fail [36]. In addition to eliminating the lesion, microfracture drilling, retrograde drilling, antegrade drilling, implanting cancellous bone grafts, osteochondral transplants, and implanting autologous chondrocytes are surgical methods of treatment [37]. However, the long-term efficacy of these treatment methods needs further investigation [38].

## 3. Regenerative Therapy in OA

Regenerative therapy is a rapidly expanding method used to treat OA because conventional therapies are ineffective and only manage to reduce symptoms and enhance function temporarily [39]. In addition, traditional therapy does not address the fundamental issue of cartilage and osteochondral bone loss [40]. MSC/stromal cell intra-articular injections improves cartilage and meniscal tissue regeneration [41, 42]. It slows the progression of OA by reducing synovial membrane inflammation, according to several preclinical studies [43, 44]. Untreated OA does not heal on its own, and existing treatments are limited due to cartilage's lack of blood supply, making stem cell therapy, particularly with MSCs, a promising option for regenerating joint tissue, with advances rapidly moving from animal models to clinical applications [45].

The hemoderivatives like PRP therapy promote anti-inflammatory and healing effects in joint disorders and soft tissue injuries, offering a beneficial alternative to corticosteroid treatments, especially in young animals or those with metabolic issues [46]. Most of the therapeutic potential in MSC therapy results from their paracrine action on host cells, mediated via cytokines, exosomes, growth factors, and extracellular matrix molecules [47, 48]. Cytokines, chemokines, trophic factors, and EVs work together to provide the angiogenic, anti-inflammatory, antioxidant, and antifibrotic activity essential for MSCs to function therapeutically [48, 49]. Even though no significant side effects are documented, cell treatments could still present some risks. These include MSCs protumorigenic properties and development into undesired cell types or tissues [50–52]. Moreover, challenges, such as cell death and leakage at the injection site must be addressed, potentially through combining MSCs with biomaterials to enhance treatment outcomes [53].

The effectiveness of stem cells for treating OA has been clinically studied. Examples include bone marrow-derived MSCs (BMSCs) and adipose-derived MSCs (AD-MSCs) [54–57]. The safety and efficacy of systemic administration of xenogeneic MSCs in dogs with OA have been documented. Low doses significantly improve clinical outcomes and cartilage structure, with a targeted effect on injury sites, making it a promising and accessible treatment for canine OA [58]. While both are commonly used with comparable efficacy, adipose tissue has been shown to contain higher concentrations of resident MSCs in their pericytes [59]. In addition, a growing number of studies have suggested that the paracrine function of stem cells, which includes the release of EVs, is primarily responsible for the therapeutic actions of stem cells [60–63].

## 4. Exosomes

The term exosomes was coined by Trams et al. [64] in 1981 to explain vesicles released by cultured cells enriched with membrane ectoenzymes. Vesicles are spontaneously secreted into the extracellular environment by most cells. EVs are lipid bilayer particles secreted by proliferating and non-proliferating cells [65]. Exosomes, microvesicles (MVs), and apoptotic bodies are the three different forms of membrane-bound EVs [66]. However, exosomes have garnered the utmost attention over the past 10 years as a key type of EV. Different cells can secrete exosomes with varied compositions of lipids, nucleic acids, and proteins to mediate intercellular communication [23]. The exosomes, having a lipid bilayer, can fuse with the target cell membrane and thus can deliver their contents, including mRNA, miRNA, DNA, proteins, and lipids [21]. Exosomes typically have a diameter between 30 and 150 nm and a density between 1.13 and 1.19 g/mL [65]. Exosomes are integral to numerous biological processes, including immune response, cell signaling, and tissue repair, due to their ability to transfer bioactive molecules between cells [67].

Ultrafiltration uses size-based filters to separate exosomes, allowing efficient processing of large sample volumes. However, it may also co-isolate particles of similar size, which can compromise the purity of the exosome preparation and potentially cause vesicle aggregation [68]. Density gradient centrifugation separates exosomes based on density through a gradient medium, often resulting in high purity and well-defined exosome populations. Despite its effectiveness, this method is time-consuming, requires specialized equipment, and may lead to some loss of exosomes during the preparation and centrifugation processes [69]. Immunoaffinity capture utilizes antibodies targeting specific exosome surface markers (e.g., CD9, CD63, and CD81) to isolate exosomes with high specificity, enriching the exosome population based on their surface proteins. However, this method can be costly and may not capture all exosome subtypes, potentially introducing bias depending on antibody specificity [70].

The primary criteria for exosome identification include morphological characteristics, particle size, and marker proteins, such as CD9, CD63, CD81, and HSP90 [71]. The features of exosomes can be determined using a variety of techniques. First, exosomes can be directly identified using transmission electron microscopy (TEM) or scanning electron microscopy (SEM). Second, exosome concentration and particle size might be examined using nanoparticle tracking analysis (NTA). NTA-based detection is a reasonably straightforward technique, and the outcome can be better quantified. Third, the western blot (WB) method aids in determining the presence of particular marker proteins in exosomes, such as CD63, TSG101, flotillin-1, ALIX, CD9, CD81, and CD82 [72]. Finally, exosome size can be determined using flow cytometry (FCM) by identifying targeted exosomes with specific antibodies or fluorescent dyes [73, 74].

## 5. Biogenesis of MSC Exosomes (MSC-Exos)

**Figure 3:**
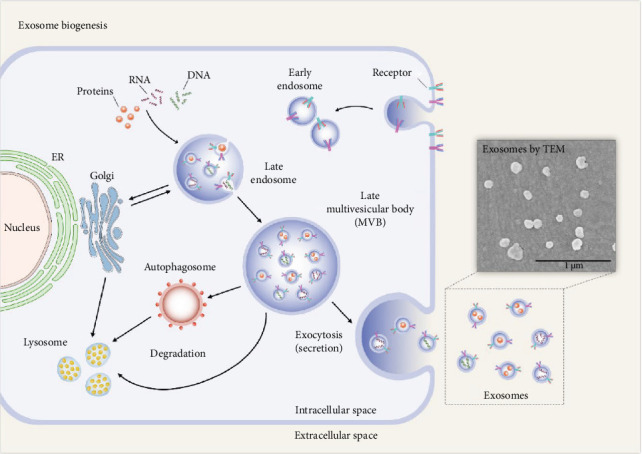
Illustrates the steps in the biogenesis of mesenchymal stem cell exosomes. The insert shows exosomes of SHSY5Y cells. Reproduced from [76] under the terms and conditions of the Creative Commons Attribution (CC BY) license (http://creativecommons.org/licenses/by/4.0/).

MSCs release exosomes into the media in which they are cultured [75]. The biogenesis and release of MSC-derived exosomes are detailed below (Figure 3). The early endosomes are formed by invagination of the cell membrane (endocytosis), which mature into late endosomes, also known as multivesicular bodies (MVBs) [77]. During this maturation process, intraluminal vesicles (ILVs) are formed within the endosomal lumen through the inward budding of the endosomal limiting membrane [78]. Thus, the large MVBs will have many ILVs with cytosolic components within them [72]. The formation of ILVs has been shown to follow either an endosomal sorting complex required for transport (ESCRT) dependent pathway or the ESCRT independent pathway. The ESCRT pathway is composed of a complex set of proteins. Four such ESCRTs have been identified, which are required for the clustering of the cargo, formation of the inward buds, and the pinching off of vesicle/membrane scission [79–81]. While ESCRT-0, ESCRT-I, and ESCRT-II assemble in the cytoplasm and participate in cargo recognition and membrane deformation, ESCRT-III is recruited to the membrane later in the process and is directly involved in vesicle scission [82]. Evidence suggests that exosome biogenesis can occur independently of the canonical ESCRT pathway [83–85]. The ESCRT-independent pathways involve heat shock proteins (HSPs), lipids, and tetraspanins [86].

To release the exosomes, MVBs will initially fuse with the cell membrane. This is mediated by Rab GTPases and SNAREs (soluble N-ethylmaleimide-sensitive factor attachment protein receptors), a family of proteins [87]. Specific members of the Rab family, such as Rab27a and Rab27b, have been implicated in regulating exosome release [74]. The interaction between vesicular SNAREs (v-SNAREs), such as VAMP (vesicle-associated membrane protein), and target membrane SNAREs (t-SNAREs) facilitates the merging of the membranes, allowing exosomes to be released into the extracellular space [57, 88, 89]. Exosomes are internalized by target cells through various mechanisms, such as endocytosis, direct membrane fusion, clathrin-mediated endocytosis, and macropinocytosis, facilitating the delivery of their cargo, including proteins and nucleic acids, thereby influencing various cellular processes [90]. Exosomes carry a variety of membrane proteins, lipids, nucleic acids, and cytosolic proteins that determine their targeting specificity and functions in recipient cells. The molecular mechanisms involved in the sorting and packaging of these cargo molecules are important in developing effective exosome-based therapies [91].

## 6. Therapeutic Effects of Exosomes in OA

Exosome helps to control the inflammatory cascades, evidenced by MSC-derived exosomes decreasing IL-1β [92]. MSC-derived exosomes reduce inflammatory mediators and NLRP3 inflammasome activation. These anti-inflammatory effects are partially mediated through adenosine receptor activation and downstream phosphorylation of protein kinase B (AKT), extracellular signal-regulated kinase (ERK), and adenosine monophosphate-activated protein kinase (AMPK) [93]. Furthermore, exosomal miR-410 from MSCs controls pyroptosis and inhibits the NLRP3 pathway [72]. It helps to revert the chondrocyte extracellular matrix to regain normal structure. BMSC-derived exosomes increased collagen type II production and decreased the expression of the MMP13 protein [94]. A systematic review of 13 preclinical studies involving 434 animals found that MSC-Exos consistently promoted repair and regeneration of osteochondral defects, with treated animals displaying increased cellular proliferation, enhanced matrix deposition, and improved histological scores [18]. In OA, exosomes derived from MSCs have demonstrated the ability to modulate inflammation and promote cartilage repair, potentially offering a non-invasive therapeutic approach to managing disease progression [95]. They can transport growth factors, cytokines, and miRNAs that influence the behavior of chondrocytes and synoviocytes, thus impacting the inflammatory and repair processes in the joint [96].

MSC-derived exosomes modulate macrophage polarization through multifaceted mechanisms involving adenosine signaling, miRNA delivery, prostaglandin E2 (PGE2) activity, and direct cytokine interactions [97]. These exosomes shift macrophages from a proinflammatory M1 phenotype to an anti-inflammatory M2 state, a critical process in mitigating inflammation and promoting tissue repair. One primary mechanism involves CD73/adenosine signaling. MSC-Exos express CD73, an ecto-5′-nucleotidase that converts extracellular adenosine monophosphate (AMP) to adenosine [97]. Adenosine binds to A2A and A2B receptors on macrophages, activating downstream AKT and ERK phosphorylation pathways [97, 98]. This signaling cascade suppresses proinflammatory cytokine production (e.g., TNF-α and IL-12) while enhancing anti-inflammatory IL-10 secretion, driving M2 polarization [17, 97]. Inhibition of CD73 or adenosine receptors abolishes this effect, confirming the pathway's necessity [97, 99]. Exosomal miRNAs are pivotal in regulating macrophage plasticity. MSC-Exos deliver miR-21-5p, which targets SPRY2, a negative regulator of ERK signaling, thereby promoting M2 polarization via the SPRY2/ERK axis [98, 100].

Similarly, miR-124-3p inhibits Ern1, reducing endoplasmic reticulum stress and inflammatory responses, while miR-125a downregulates IRF5, a transcription factor critical for M1 activation [101, 102]. miR-223, another exosomal cargo, suppresses C/EBP*β* and PDCD4, disrupting proinflammatory signaling and enhancing M2-associated anti-inflammatory responses [17, 102]. These miRNAs act synergistically to repress M1-related genes and activate M2 transcriptional programs. PGE2-mediated pathways further contribute to immunomodulation. MSC-Exos enriched with PGE2 activate STAT6 and mTOR signaling in macrophages, upregulating M2 markers like Arg1 and Ym1 [17, 99]. Pharmacological inhibition of COX-2, a key enzyme in PGE2 synthesis, ablates this polarization, underscoring PGE2′s role [99]. Additionally, mTOR activation enhances glucose metabolism and IDO1 activity, fostering an anti-inflammatory microenvironment conducive to M2 differentiation [17, 99]. Surface proteins and cytokines within exosomes directly engage macrophage receptors. MSC-Exos carry TGF-β and IL-10, which bind to cognate receptors on macrophages, initiating Smad and STAT3 signaling to suppress NF-κB-driven inflammation [101]. Exosomal EDA-fibronectin activates Toll-like receptor (TLR) pathways, synergizing with adenosine to reinforce M2 polarization [17, 97]. Furthermore, exosomes transfer functional CD73 to macrophages, amplifying adenosine production and creating a feedforward loop that sustains M2 dominance [98]. These mechanisms collectively enable MSC-Exos to reprogram macrophage metabolism and gene expression, resolving inflammation and facilitating tissue repair. By integrating adenosine signaling, miRNA delivery, lipid mediators, and cytokine interactions, MSC-Exos offer a multifaceted approach to modulating immune responses in inflammatory diseases.

Exosome helps improve chondrogenic differentiation, evidenced by MiR-8485 from exosomal chondrocytes triggering the Wnt/-catenin pathways to promote BMSC development into chondrocytes [103]. The enrichment of therapeutic molecules in MSC-derived exosomes is primarily accomplished through overexpression of various noncoding RNAs, including miRNAs, long noncoding RNAs (lncRNAs), and circular RNAs (circRNAs), with extensive evidence supporting that miRNAs can significantly promote exosome-mediated cartilage regeneration [104]. EV cargo, such as miRNA, can encourage chondrocyte growth [105–107]. Specifically, miR-92a-3p-overexpressing BMSC-derived exosomes have been shown to promote cartilage proliferation and reduce cartilage matrix synthesis by targeting WNT5A and inhibiting the WNT signaling pathway [108]. EV prevents apoptosis and autophagy. Research has demonstrated that exosomes derived from miR-100-5p-rich MSCs reduce chondrocyte apoptosis and ECM degradation through mTOR signaling in vitro, while in vivo studies show these exosomes maintain cartilage homeostasis, reduce chondrocyte apoptosis, promote ECM synthesis, and improve the poor gait of surgically induced OA mice [109, 110]. Research has demonstrated the value of EVs in preventing endoplasmic reticulum-induced apoptosis in intervertebral disc (IVD) degeneration [111, 112]. In brief, the basic mechanism of action of exosomes in OA is the restoration of cartilage homeostasis. BMSC exosomes have the potential to influence a variety of cell fates, including death, proliferation, invasion, and migration. The immunological response, osteogenesis, fibrosis, and angiogenesis are just a few of the physiological and pathological processes that BMSC-derived exosomes can control [94]. Exosomes produced by BMSCs have been shown in numerous studies to aid significantly in regenerating and restoring damaged tissues, including cartilage and subchondral bone [94, 103]. For example, intra-articular injection of BMSC exosomes decreased articular cartilage degeneration and subchondral bone deterioration in the collagenase-induced mouse model. Similarly, BMSC exosomes can substantially reverse cartilage and subchondral bone damage in the lumbar facet joint OA model [103].

Exosomes produced from synovial MSCs have recently been shown to promote cartilage regeneration and successfully slow OA progression [113]. Synovial MSC (SMSC)-derived exosomes could stimulate articular chondrocyte proliferation and emigration in a Wnt5a/Wnt5b/YAP-dependent way. Additionally, therapy with SMSC-140-Exo markedly reduced joint wear, lowered OARSI scores, and slowed the course of OA in a rat OA model [114]. A good cell source for treating OA is AD-MSC, which has been shown to have a strong capacity for cartilage regeneration and inflammation control. EVs, such as MVs and exosomes, play a major role in mediating the paracrine effects of AD-MSC on osteoarthritic osteoblasts [115].

Embryonic MSCs have also been proposed as a potential treatment for OA and cartilage repair. Exosomes derived from embryonic MSCs have been demonstrated to alleviate OA by balancing the synthesis and degradation of the cartilage extracellular matrix, promoting chondrocyte proliferation and enhancing matrix synthesis while inhibiting catabolic pathways [116]. They have recently been found to modulate chondrocyte biological characteristics and slow the onset of OA in experimental OA models [117, 118]. Furthermore, embryonic stem cell-derived MSC-Exos have shown the ability to repair OA through suppression of pain, reduced inflammation, and gradual improvements in matrix expression [119]. The amniotic fluid stem cells (AFSCs) exosomes increased pain tolerance and nearly fully and regularly regenerated hyaline cartilage [120]. Umbilical MSC-Exos (U-MSC-Exos) have shown potent chondroprotective benefits in vivo cartilage repair [121]. Exosomes derived from human umbilical cord MSCs rich in miR-100-5p have been shown to inhibit ROS production and cell apoptosis induced by cyclic tension by directly targeting NADPH oxidase 4 (NOX4) [110]. In addition, it has been found that the exosomes of stem cells from human exfoliated deciduous teeth (SHEDs) minimize inflammatory responses and maintain anabolism balance in chondrocytes of the temporomandibular joint [122, 123].

In a study using an ovine sheep model of low-grade OA, combined intra-articular injections of AD-MSC-Exos and HA were shown to delay disease progression [124], with the combination therapy resulting in significantly lower lameness scores than HA or exosome treatments alone. However, while radiographic improvements were observed, macroscopic evaluations did not show significant differences, and longer follow-up is needed to assess the long-term effects of this combined therapy [124]. Current research indicates that MSC-derived exosomes have demonstrated strong anti-inflammatory qualities that can reduce chronic inflammation, stimulate chondrocyte proliferation, and modulate immune responses, with promising results from preclinical studies showing effective therapeutic responses in OA treatment with minimal side effects [125]. The inflammatory regulatory capacity of exosomes varies substantially among different subpopulations, representing a critical knowledge gap that influences therapeutic efficacy in OA treatment. Previous investigations have revealed distinct protein expression patterns across exosome subpopulations [126]. This subpopulation heterogeneity extends to inflammatory regulation. Furthermore, the cellular origin of exosomes significantly determines their inflammatory modulatory effects, with MSC-derived exosomes consistently demonstrating anti-inflammatory properties [127]. The differential cargo composition, including varying concentrations of miRNAs, proteins, and metabolites among subpopulations, directly influences their therapeutic potential and necessitates precise characterization for optimal clinical applications [21].

## 7. Exosomes in Canine and Equine OA

Exosome-based therapy is an emerging field in veterinary medicine, and its therapeutic applications are mostly reported in horses and dogs [128, 129]. The EVs, including exosomes, are prime mediators of MSCs' function as they are the key component of the MSC-secretome [130]. The exosomes derived from canine BMSC and AD-MSC were the first to be characterized [131]. Their study used TEM to assess the size and shape, and WB to detect the specific surface protein markers of exosomes [131]. Their study reported that BMSCs produced 13 times more exosomes than AD-MSCs. The proregenerative properties of MSCs are attributed to their secretory factors, including the exosomes they secrete [132].

The exosomes derived from MSCs could be used as its substitute due to their regenerative and anti-inflammatory properties, which offer the additional advantage of using a cell-free product that reduces tumorigenic potential and immunologic reactions that would be otherwise induced by the parent cells [128, 129, 133, 134]. The effect of xenogenic pure exosome product (PEP) on canine tenocytes was investigated in vitro. It was found to exert a positive regulatory role on canine tenocytes, indicated by enhanced cell proliferation, migration, and collagen deposition [135]. In addition, the effect of freeze-dried EVs (secretome) derived from canine AD-MSC on spontaneous arthritis in dogs was studied by Mochi et al. (2021) [136 ]. Their study characterized the secretome and was conducted in vivo in dogs with no progression in lameness [136].

Orthobiologics in veterinary medicine is mainly associated with the equine practice. OA is a common musculoskeletal disorder in horses, mainly associated with welfare and economic issues. Several studies on the therapeutic effect of orthobiological preparations, like PRP, autologous conditioned serum (ACS), stem cell therapy, etc., have been reported in horses [137, 138]. The use of stem cell therapy to treat equine orthopedic cases has recently become widespread. MSCs exert their paracrine action by producing regenerative and anti-inflammatory substances in membrane-bound EVs (exosomes, MVs, and apoptotic bodies). The main advantage of using exosomes is in the ease of production, usage, storage, and standardization using the expression of specific membrane surface protein markers like CD9, CD63, and CD81, which are important for their formation and transportation within the cells and their target cells [139].

Exosomes derived from equine AD-MSC were isolated through ultracentrifugation, ultrafiltration, and charge-based precipitation methods and characterized through NTA, immunohistochemistry, WB, immunoelectron microscope, and TEM [140]. Their study proposed a therapeutic potential of equine AD-MSC-derived exosomes in veterinary medicine. Similarly, the EVs from equine BMSC through ultracentrifugation were isolated and characterized [141]. Further, their effect on equine chondrocytes cultured in vitro in a proinflammatory environment mediated by IL-1β and TNF-α was investigated. Their study concluded that equine chondrocytes internalize EVs and exhibit anticatabolic action when cultured with proinflammatory cytokines. The therapeutic potential of exosomes as prominent mediators of homeostasis, repair, and regeneration has to be explored. The impact of exosomes derived from equine BMSCs on equine articular chondrocytes (eACs) was evaluated using different purification methods and proinflammatory cytokine priming [142]. The researchers found that exosomes purified by membrane affinity capture (MAC) enhanced the production of a hyaline-like matrix in eACs more effectively than those purified by size-exclusion chromatography (SEC) [142]. Additionally, MSC-exosomes primed with proinflammatory cytokines, such as IL-1β and TNF-α, showed even greater improvement in matrix synthesis, highlighting the significance of both purification techniques and cytokine priming in optimizing MSC-exosome therapies for equine OA [142].

**Figure 4:**
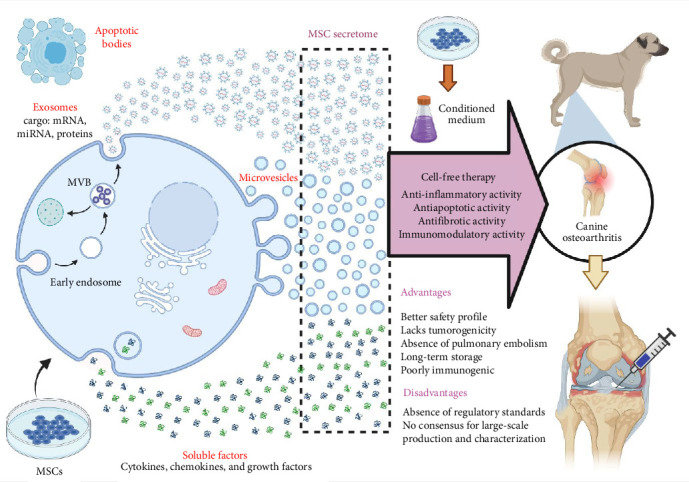
Overview of cell-free therapy using mesenchymal stem cell-derived extracellular vesicles in canine osteoarthritis.

Exosome-based therapy could be a promising tool in the field of veterinary orthopedics in the future (Figure 4 and Table 1). The therapeutic effects of exosomes have been demonstrated in various preclinical studies, with BMSCs, AD-MSCs, and other stem cell sources showing promise in regenerating and restoring damaged tissues, including cartilage and subchondral bone. These studies provide valuable insights into the potential clinical applications of exosome-based therapies for OA. Moreover, exploring exosomes in canine and equine OA offers a glimpse into the future of veterinary regenerative medicine. Exosomes derived from MSCs in these species demonstrate positive regulatory roles on cells, enhanced cell proliferation, migration, collagen deposition, and potential therapeutic effects in spontaneous arthritis in dogs (Tables 1 and 2). In equine orthopedics, the characterization and therapeutic potential of exosomes derived from equine AD-MSCs and BMSCs open new avenues for addressing musculoskeletal disorders in horses. Despite these promising findings, several challenges and questions remain.

## 8. Limitations and Challenges in Veterinary Medicine

While stem cell-derived exosomes offer a promising therapeutic approach for OA in veterinary medicine, several limitations must be acknowledged [146]. First, the current body of research on exosome therapy in veterinary species remains limited compared to human studies. Most of the available data are preclinical, and significant gaps exist regarding the safety, efficacy, and standardization of exosome-based treatments in animals. Additionally, the heterogeneity of exosomes derived from different stem cell sources presents a challenge in defining consistent therapeutic protocols. Variability in isolation methods, characterization techniques, and dosing regimens further complicates direct comparisons across studies, making it difficult to establish clear guidelines for clinical use. Another key limitation is the lack of long-term studies assessing the durability of therapeutic effects and potential side effects in veterinary patients.

While MSC-derived exosomes have demonstrated disease-modifying effects, their therapeutic responses can vary depending on the cell source. OA affects the entire joint, and utilizing exosomes from multiple cell sources may offer an alternative strategy. However, the extracellular matrix of articular cartilage poses a challenge, with pore sizes around 6.0 nm acting as a biological barrier [147]. Since exosomes range from 30–150 nm in size, enhancing their delivery into chondrocytes is crucial [148]. Additionally, the thickness of cartilage limits exosome permeability, making it difficult for them to reach subchondral progenitor cells [149]. Encapsulating exosomes in scaffolds could provide controlled release, reducing the need for frequent injections, but the material properties and pharmacokinetics of such scaffolds require further investigation [150]. Although preclinical studies have shown promise, translating these findings into effective OA treatments for both humans and animals remains challenging [151]. A major obstacle is the lack of standardized protocols for exosome isolation, characterization, and administration, which hampers reproducibility and comparability across studies. Additionally, large-scale exosome production is limited by issues, such as short circulation time, suboptimal targeting, and inadequate control mechanisms [152].

The development of OA treatments in veterinary medicine faces unique anatomical and biomechanical challenges compared to human medicine. Joint architecture and cartilage thickness vary significantly across species, influencing treatment efficacy and delivery strategies. For example, equine articular cartilage thickness (1.5–2 mm) closely approximates human cartilage (2.2–2.5 mm), making horses valuable preclinical models for cartilage repair studies [153]. In contrast, canine cartilage thickness (0.6–1.3 mm) and feline cartilage (often <0.5 mm) present distinct barriers for preclinical research [153]. Additionally, joint mechanics differ markedly between quadrupeds and humans. In dogs, joint instability due to cranial cruciate ligament injuries or hip dysplasia exacerbates OA progression, while in horses, repetitive loading during athletic activity drives subchondral bone remodeling and synovitis [154, 155]. These species-specific biomechanical stressors necessitate tailored therapeutic approaches, such as targeted anti-inflammatory strategies for equine OA or stability-restoring interventions for canine cruciate injuries [154, 155].

Regulatory frameworks for veterinary biologics differ substantially from human therapeutics, creating hurdles for exosome-based treatments [156]. Exosome therapies may face classification ambiguities, depending on whether they are deemed biologics or drugs [157]. Veterinary regulatory processes often lag behind, with limited standardized protocols for exosome characterization, dosing, or safety testing. Ethical considerations, such as minimizing animal distress during repeated intra-articular injections, further complicate clinical translation [158]. Exosome delivery in veterinary patients presents logistical and physiological challenges. Intra-articular injection remains the primary method, but anatomical accessibility varies: equine joints require specialized techniques for accurate placement, while canine and feline joints are smaller and more prone to injection-related trauma [159]. The avascular nature of cartilage and the rapid clearance of exosomes from synovial fluid limit sustained therapeutic exposure, necessitating frequent dosing or encapsulation strategies [160]. Cartilage thickness also impacts exosome distribution. In horses with thick cartilage, exosomes may fail to reach subchondral progenitor cells, reducing regenerative potential [161]. Scaffolds or bioengineered carriers could enhance retention, but material biocompatibility and cost-effectiveness remain unresolved issues in veterinary practice. Additionally, species-specific immune responses to allogenic exosomes necessitate further research to ensure safety and efficacy [162].

OA diagnosis and treatment monitoring in veterinary medicine face unique obstacles. Unlike humans, animals often mask pain, delaying intervention [163]. For example, felines may exhibit behavioral changes (e.g., reduced grooming and altered sleep patterns) rather than overt lameness, complicating early detection [163]. Imaging modalities like MRI are less accessible in veterinary settings, relying instead on radiography or clinical scoring systems with limited sensitivity. Post-treatment monitoring also differs. In equine OA, lameness scores and gait analysis are critical, whereas canine OA relies on owner-reported quality of life metrics [164]. The lack of standardized biomarkers for OA progression or exosome efficacy hampers objective assessment, particularly in species with subtle clinical signs [165]. Treatment goals diverge between veterinary and human OA management. In companion animals, preserving quality of life and minimizing pain take precedence over disease modification, whereas human therapies increasingly target cartilage regeneration [166]. In contrast, human trials prioritize cartilage repair mechanisms, such as exosome-mediated chondrogenesis.

Advanced isolation technologies and quality control measures can drive up the cost of exosome production, potentially limiting their availability in routine veterinary care. Overcoming these limitations will require concerted efforts in research, standardization, and clinical trials to translate the potential of exosome therapy from bench to bedside in veterinary medicine. Despite existing challenges and limitations, the future of exosome-based therapies for OA is bright. Continued research, focusing on safety and standardized protocols, could revolutionize OA treatment in both human and veterinary medicine.

## 9. Future Prospects of MSC-Derived Cell-Free Therapy in OA

Recent advancements in exosome engineering can overcome current limitations in cartilage penetration and biodistribution [22]. By modifying surface proteins or glycans, exosomes can be tailored to target chondrocytes or synovial cells more effectively [22]. For instance, a recent study demonstrated that chondrocyte-affinity peptide (CAP)-functionalized exosomes, when combined with liposomal CRISPR-Cas9 systems, successfully suppressed MMP-13 expression in osteoarthritic chondrocytes and penetrated deep into cartilage matrices in rat models [167]. Similarly, enzymatic glycoengineering techniques enable the addition of azide tags to exosomal glycans, allowing precise attachment of ligands (e.g., antibodies against collagen type II) via click chemistry [168]. These modifications enhance retention within joints while minimizing systemic clearance, addressing the challenge of rapid exosome elimination observed in canine and equine studies.

Combining exosomes with hydrogels or cryogels is emerging as a strategy to prolong therapeutic effects and reduce dosing frequency [169]. The combination of primary chondrocyte-derived exosomes with a thermosensitive hydrogel enables sustained intra-articular delivery, enhancing their retention at the cartilage site [170]. This combination approach has the potential to outperform standalone exosome injections. These scaffolds not only provide mechanical support but also mimic native cartilage microenvironments, enhancing exosome-mediated repair [169].

Exosomes are also being harnessed as versatile and biocompatible vehicles for delivering gene-editing tools, such as CRISPR/Cas9 systems or synthetic miRNA/siRNA, aimed at modulating key molecular pathways involved in OA pathogenesis [167]. These targeted approaches hold promise for correcting dysregulated gene expression associated with cartilage degradation, inflammation, and abnormal immune responses [171]. Looking ahead, future therapeutic protocols may incorporate personalized miRNA cocktails tailored to individual animals, based on synovial fluid biomarker profiling [171]. This would enable a precision medicine approach in veterinary OA management, particularly for predisposed breeds like Labradors and German Shepherds, where genetic susceptibility and biomechanical factors contribute significantly to early-onset or severe OA [172]. Such strategies could help optimize treatment outcomes by addressing the molecular underpinnings of disease in a breed-specific and patient-specific manner.

The applicability of different exosome isolation methods in OA research remains a critical consideration, as methodological variability directly impacts exosome yield, purity, and functional properties. Addressing the heterogeneity in exosome isolation remains a critical challenge for the clinical translation of exosome-based therapies. Traditional ultracentrifugation, although widely used, often yields preparations with low purity and co-isolated contaminants, such as protein aggregates and lipoproteins [173]. In contrast, SEC achieves higher purity but may exclude smaller exosome subsets critical for chondrocyte communication [174]. Precipitation-based methods, though scalable, risk polymer contamination that could alter exosome surface properties and targeting efficiency [174]. These findings underscore the need for method-specific validation in OA models, particularly as isolation techniques influence exosome biodistribution in articular tissues. Recent advances in charge-based precipitation methods and membrane affinity chromatography have demonstrated improved yield and purity, allowing for more standardized and scalable isolation suitable for therapeutic applications [173, 175]. These methods offer a more reproducible approach and are increasingly integrated into good manufacturing practice (GMP)-compliant workflows [176]. Furthermore, the emergence of microfluidic platforms and tangential flow filtration (TFF) systems is facilitating high-throughput, large-scale production of exosomes with minimal batch-to-batch variability [176–178]. Microfluidic chip-based isolation has emerged as a transformative approach for exosome purification, addressing critical limitations of conventional methods through miniaturized fluidic systems that enhance precision, efficiency, and integration with downstream analytical workflows [176]. These technologies not only reduce processing time and costs but also enhance the ability to isolate functionally intact and size-homogeneous exosomal populations.

Species-specific exosome profiling is another important area of progress [179]. Comparative studies are beginning to identify unique surface markers and cargo profiles, such as miRNA, lncRNA, and proteins, that vary significantly between species and cell sources [179, 180]. This profiling is crucial for optimizing therapeutic efficacy in veterinary settings, as it helps tailor exosome preparations to the unique pathophysiology of different animals. In clinical practice, these insights may soon allow veterinarians to select exosome sources based on the underlying etiology of OA [181]. For instance, exosomes for post-traumatic OA may require cargo that promotes chondrogenesis and immune modulation, while those targeting dysplasia-associated OA may prioritize anti-inflammatory or cartilage-protective molecules [182]. Integration of patient-specific biomarkers derived from synovial fluid or circulating cytokine profiles could enable a more personalized and precision-based therapeutic approach [183].

Safety remains a central concern in exosome therapy. While early trials in dogs have reported no signs of tumorigenicity, immunogenicity, or adverse tissue responses, these results are preliminary (Table 1). While MSC-derived exosomes also demonstrate favorable safety profiles in preclinical OA models, dose-dependent effects require rigorous characterization [18]. Longitudinal studies with extended follow-up periods are essential to evaluate delayed or cumulative risks, including ectopic tissue formation, chronic inflammation, or immune sensitization. The therapeutic window for exosome applications in OA requires careful evaluation of dose-dependent effects, as current safety data reveals complex relationships between dosage and biological responses. Toxicological evaluations have demonstrated that AD-MSC-Exos exhibit favorable safety profiles across multiple testing parameters, including skin sensitization, eye irritation, and acute oral toxicity assessments, with no adverse effects observed in comprehensive testing protocols [184]. However, dose-dependent variations in therapeutic efficacy have been documented, with studies reporting effective doses ranging from micrograms to hundreds of micrograms per application, suggesting that optimal dosing remains highly context-dependent [185]. The complexity of dose optimization is further compounded by the observation that while lower exosome concentrations may facilitate certain cellular responses, such as phagocytosis, higher concentrations can exhibit diminished effects or potentially trigger different biological pathways [185, 186]. This emphasizes the critical need for systematic dose-escalation studies and long-term safety monitoring to establish therapeutic windows that maximize beneficial effects while minimizing potential adverse reactions.

Artificial intelligence (AI)-driven platforms are being developed to analyze the molecular cargo of exosomes in depth [187]. These platforms can identify predictive biomarkers within the cargo, such as specific miRNAs or proteins associated with positive treatment outcomes [188]. This data-driven approach has the potential to stratify patients into likely responders and nonresponders before therapy is initiated, thereby improving clinical outcomes and reducing unnecessary costs, especially in refractory cases [189]. Collectively, these technological and translational advances are paving the way for a new generation of safe, effective, and customizable exosome-based therapies for OA in both humans and veterinary patients [187, 188].

The future landscape of exosome-based OA therapy requires integration with emerging biotechnological platforms and precision medicine approaches to address current limitations and enhance therapeutic efficacy. Advanced engineering strategies, including the development of targeted exosome delivery systems using biocompatible materials, such as hydrogels and cryogels, represent promising avenues for improving therapeutic outcomes through enhanced tissue penetration and sustained release mechanisms [182]. These technological advances, combined with improved isolation standardization and comprehensive safety profiling, position exosome therapy as a potentially revolutionary approach for addressing the complex pathophysiology of OA while overcoming current therapeutic limitations.

## 10. Conclusions

OA remains a prevalent and debilitating joint disease, necessitating effective and economical treatment strategies. The multifactorial nature of OA, influenced by factors such as age, obesity, mechanical injuries, and joint trauma, presents a complex challenge for both researchers and clinicians. Current therapeutic approaches, including pharmacologic analgesia and surgical interventions, face limitations, such as source availability, graft rejection risks, and the development of fibrocartilage rather than hyaline cartilage. In this context, regenerative therapy, particularly using MSC-derived exosomes, emerges as a promising avenue. These exosomes play a crucial role in mediating the paracrine effects of MSCs, offering potential benefits in cartilage regeneration and inflammation control. The mechanism of action involves the modulation of inflammatory cascades, suppression of proinflammatory mediators, and promotion of chondrogenic differentiation. The therapeutic effects of exosomes in OA have been extensively studied, with promising results in preclinical models. Exosomes have shown efficacy in reducing articular cartilage degeneration, subchondral bone deterioration, and overall disease progression in experimental models of OA. The application of exosomes in veterinary medicine, particularly in canine and equine OA, is a rapidly evolving field. Exosome-based therapies derived from MSCs of canine and equine origin have shown promise in promoting regenerative and anti-inflammatory effects. These findings suggest a potential shift towards cell-free products, such as exosomes, as viable alternatives to traditional cell-based therapies in veterinary orthopedics.

Harnessing EVs, particularly exosomes, holds immense potential as a cell-free therapeutic approach for OA in veterinary medicine. As research advances, exosome-based therapies are anticipated to significantly improve the quality of life for animals affected by OA. Exploring exosomes in veterinary medicine benefits our animal companions and provides valuable insights that may inform advancements in human OA treatment.

## Figures and Tables

**Table 1:** Overview of in vivo canine studies on the therapeutic use of exosome-derived treatments for osteoarthritis.

Species	Treatment	Protocol	Main findings	Reference
Canine (labrador retriever)	Conditioned medium from allogeneic adipose tissue-derived MSCs (CM-AD-MSCs)	Intra-articular injection into both elbow joints on days 0 and 14	Significant reduction in MMP-3, TIMP-1, IL-6, and TNF-α levels in synovial fluid post-treatment. Considerable improvement in range of motion was observed from day 0 to 42. No severe adverse events were reported, supporting the safety and efficacy of CM-AD-MSC for OA management	[143]

Canine (dog)	Canine MSC-secretome (lyosecretome)	Intra-articular injections	In vivo, two intra-articular injections were administered to dogs with bilateral knee or elbow osteoarthritis. Follow-up showed no clinically significant local or systemic adverse effects, indicating the treatment's safety	[136]

Canine (dog)	Blood cell secretome (BCSG), triamcinolone (TG), and combination (BCS + TG)	Intra-articular injections: 3 mL BCSG, 0.5 mL TG, or 3.5 mL combination	The BCS + TG group showed significant improvement in pain scores from days +8 to +150. The combination also yielded better outcomes in canine orthopedic index dimensions, with the Kaplan–Meier analysis indicating long-lasting benefits in the BCS + TG group, followed by BCSG and TG	[144]

**Table 2:** In vitro studies on the effects of exosome-derived treatments in cartilage tissue.

Species	Exosome source	Methodology	Main findings	Reference
Canine (dog)	Canine MSC-secretome (lyosecretome)	Freeze-dried lyosecretome prepared according to ISO9001 standards	Lyosecretome induced dose-dependent proliferation of canine MSCs, tenocytes, and chondrocytes, and exhibited 85% antielastase activity at 20 mg (mL)	[136]

Equine (horse)	Bone marrow-derived mesenchymal stem cells (BMSCs)	Membrane affinity capture (MAC), size-exclusion chromatography, proinflammatory cytokine priming	MSC-derived exosomes (MSC-Exos) were internalized by equine articular chondrocytes (eACs). MAC-exos enhanced eAC hyaline-like matrix production, but required ultrafiltration due to MAC buffer effects. Cytokine-primed MSC-Exos (especially IL-1β and TNF-α) further improved eAC hyaline-like phenotype	[142]

Equine (horse)	BMSCs	Indirect co-culture and MSC-conditioned media	MSC secretome increased cartilage functionality markers and cell migration in equine articular eACs, suggesting the potential to delay OA progression. The presence of nanosized EVs, such as exosomes, was confirmed via nanoparticle tracking assay and TEM	[145]

## Data Availability

The data sharing is not applicable to this article as no datasets were generated or analyzed during the current study.

## Nomenclature

ACS: Autologous-conditioned serum
AD-MSCs: Adipose-derived mesenchymal stem cells
AFSCs: Amniotic fluid stem cells
AKT: Protein kinase B
ALIX: ALG-2-interacting protein X
AMP: Adenosine monophosphate
AMPK: Adenosine monophosphate-activated protein kinase
BMSCs: Bone marrow-derived mesenchymal stem cells
CAP: Chondrocyte-affinity peptide
CD: Cluster of differentiation
circRNAs: Circular RNAs
COX-2: Cyclooxygenase-2
CRISPR: Clustered regularly interspaced short palindromic repeats
ECM: Extracellular matrix
EDA: Extra domain A (of fibronectin)
ERK: Extracellular signal-regulated kinase
ESCRT: Endosomal sorting complex required for transport
EVs: Extracellular vesicles
FCM: Flow cytometry
HA: Hyaluronic acid
HSP: Heat shock protein
HSP90: Heat shock protein 90
IDO1: Indoleamine 2,3-dioxygenase 1
IL: Interleukin (e.g., IL-1β, IL-10, IL-12)
ILVs: Intraluminal vesicles
IVD: Intervertebral disc
lncRNAs: Long noncoding RNAs
miRNA: MicroRNA
miRNAs: MicroRNAs
MMP: Matrix metalloproteinase
MSC: Mesenchymal stem cell
mTOR: Mechanistic target of Rapamycin
MVBs: Multivesicular bodies
MVs: Microvesicles
NLRP3: NOD-, LRR-, and pyrin domain-containing protein 3
NOX4: NADPH oxidase 4
NTA: Nanoparticle tracking analysis
OA: Osteoarthritis
OARSI: Osteoarthritis Research Society International
PEP: Pure exosome product
PGE2: Prostaglandin E2
PRP: Platelet-rich plasma
ROS: Reactive oxygen species
SEC: Size-exclusion chromatography
SHEDs: Stem cells from human exfoliated deciduous teeth
siRNA: Small interfering RNA
SMSC: Synovial mesenchymal stem cell
STAT3: Signal transducer and activator of transcription 3
TFF: Tangential flow filtration
TEM: Transmission electron microscopy
TGF-β: Transforming growth factor beta
TLR: Toll-like receptor
TNF-α: Tumor necrosis factor alpha
t-SNARE: Target membrane SNARE
U-MSC-Exos: Umbilical MSC exosomes
VAMP: Vesicle-associated membrane protein
WB: Western blot
WNT: Wingless/integrated (signaling pathway)
YAP: Yes-associated protein.
